# Behavioral Change in Determinants of the Choice of Fuels amongst Rural Households after the Introduction of Clean Fuel Program: A District‐Level Case Study

**DOI:** 10.1002/gch2.202000004

**Published:** 2020-10-26

**Authors:** Vaishali Bhole Jaiswal, Pravin U. Meshram

**Affiliations:** ^1^ Department of Environmental Science SMM Rashtrasant Tukdoji Maharaj Nagpur University Nagpur 440033 India; ^2^ Department of Epidemiology National Institute of Health & Family Welfare New Delhi 110067 India

**Keywords:** biomass fuel, cost of fuel, education, household air pollution, income, socioeconomic factors

## Abstract

Consumption of energy is a determinant of the socioeconomic status of many citizens across the globe. The majority of rural households in India are dependent on biomass fuels. Existing data on the factors affecting fuel switching in rural India are insufficient to analyze a behavioral change among families. This paper evaluates the influence of four variables income, education, cost of fuel, and clean fuel supply on fuel adoption decisions. To understand the study population's behavioral change, a Household Survey is conducted in 20 villages (in India's rural district). Along with field observation, data are also collected on energy usage at the household level using a formal questionnaire. Binary logistic regression is applied to establish a link between the variables. Both biomass fuels and Liquefied Petroleum Gas are used mostly for cooking. The prevalence of energy stacking behavior is observed even among middle and upper‐income families. Modest evidence for the “energy ladder” hypothesis is seen, however, a “switch over” to cleaner fuels is not.

## Graphical abstract

This paper evaluates the influence of four variables, i.e., income, education, cost of fuel, and clean fuel supply/security on fuel adoption decisions. To understand the study population's behavioral change, a Household Survey is conducted in India's rural district. Binary logistic regression is applied to establish a link between the variables. Primarily both biomass fuels and LPG are used mostly for the cooking. The prevalence of energy stacking behavior is observed even among middle‐ and upper‐income families.

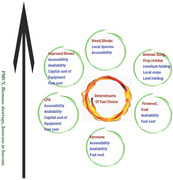

## 1 Introduction

Household air pollution (HAP) is an acknowledged health risk to exposed populations. The primary sources of HAP in developed countries are environmental tobacco smoke, volatile organic compounds from furnishings, and radon from soil^[^ ^1^ ^]^ whereas in the developing countries pollutants are released during the combustion of solid fuels, including biomass (wood, dung, and crop residues) or coal. In India 86.7% of rural and 26.3% of urban households use solid biomass fuels for their cooking needs^[^ ^2^ ^]^ adversely impacting the respiratory health of individuals and contributing to climate change.^[^ ^3^, ^4^, ^5^, ^6^, ^7^, ^8^, ^9^ ^]^ Biomass burnt in stoves (traditional Indian Chulah^1^) emits toxic pollutants like respirable particles, carbon monoxide, oxides of nitrogen and sulfur, benzene, formaldehyde, 1,3‐butadiene, and polyaromatic hydrocarbon compounds, such as benzo[α]pyrene^[^ ^4^ ^]^ which together with poor ventilation in rural households exposes the women to risk several times higher than the approved limits.^[^ ^10^ ^]^ Thus, the need for clean energy is being emphasized all over the world.

There is extensive information in the literature about household cooking energy requirements for developing countries.^[^ ^11^ ^]^ Socio‐economic status of the households determines their energy utility.^[^ ^12^, ^13^ ^]^ Households maximize their energy utilities according to their status.^[^ ^14^ ^]^ According to the energy ladder theory, the transition of household's fuel choice from traditional biomass fuels to modern is determined by its increasing income. This switching is called “Energy transition.”^[^ ^15^, ^16^ ^]^ The “energy ladder” hypothesis was supported by research studies.^[^ ^17^, ^18^, ^19^, ^20^ ^]^ Recent national surveys also support the hypothesis in India.^[^ ^21^, ^22^ ^]^ Some experts, however, feel the energy ladder theory does not describe the full dynamics of household fuel usage and the choice of fuel depends on many other factors. Patterns of multiple fuel use are common in developing countries.^[^ ^23^ ^]^ The fuel‐stacking concept suggests that the households combine different energy sources, which are not mutually exclusive, for different end‐uses, and using various combination of fuels at any given time point. In addition to income, economic, cultural, and social preferences may be equally important.^[^ ^24^ ^]^ Economic growth along with environmental pressure, technological advancement, resource availability, level of urbanization, and living standards also play an important role.^[^ ^25^ ^]^ Cooking habits, regional and cultural taste, price, availability of supply, educational status, and household composition were found to be the other important factors. The energy ladder hypothesis did not include these factors.^[^ ^26^ ^]^ Therefore, instead of moving up the ladder step by step as income rises, households choose different fuels.^[^ ^27^ ^]^ They choose a combination of high‐cost and low‐cost fuels, depending on their budgets, options, and needs.^[^ ^28^ ^]^ It is called fuel stacking (multiple fuel use), instead of fuel switching or energy ladder.^[^ ^23^ ^]^ Previously, all research studies were undertaken on the choice of energy before the launch of a major clean fuel program in India.^[^ ^29^ ^]^

The improved cooking stoves program led to the installation of 33.8 million improved stoves across the country. Biomass cooking stoves have not progressed to the point that they are equivalent to liquefied petroleum gas (LPG) in terms of reliability, flexibility, durability, efficiency, and cleanliness. Millions of women have changed from biomass to LPG when they had an option, however, millions do the reverse every year when given a chance.

Clean cooking fuels are a highly cost‐effective health intervention^[^ ^30^ ^]^ and household's energy‐behavior also indicates the economic development of a country. In “Pradhan Mantri Ujjwala Yojana” (PMUY), the government provided gas connections to a total of 50 million poor households (from 2016 to 2018).^[^ ^31^ ^]^ Piped natural gas (PNG) connections have reached more than 11% of households annually with a goal of 20 million by early next decade ^[^ ^32^ ^]^ helping to move LPG to rural areas.

Yet there is limited data available for factors determining the choice of fuels such as local availability, transport cost, and health effects involved in the biomass and clean fuels in the rural areas. A systematic study with a regional approach (after the launch of clean fuel program) was thus undertaken factoring the diversity of diet, cooking habits, and economic factors.^[^ ^33^ ^]^

## 2 Methodology

**Figure 1:**
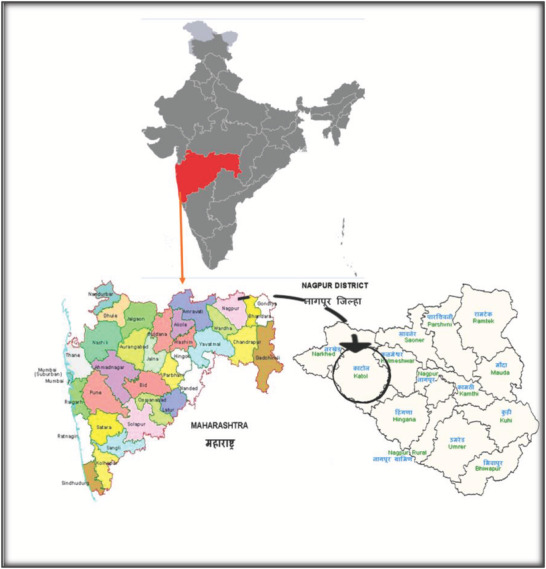
Location of the study area.

Study area was rural Nagpur district in the state of Maharashtra, India. Time of study was in the years 2016–18 (March 2016 to March 2018). Type of study was cross‐sectional with multi‐stage random sampling. District Nagpur was randomly selected from the districts^2^ of Maharashtra. Katol was selected randomly from 14 blocks^3^ in the district. Considering the distance from the highway as a factor influencing the pollution, the villages were divided into three groups; less than 10 km from the highway, 10–20 km and more than 20 km from the highway. A third of the villages in each group was selected randomly. This resulted in the selection of unequal number of villages based on the distance from the highway. The study population was rural women who cooked food (**Figure** **1**).

The prevalence of chronic obstructive pulmonary disease (COPD) in the state of Maharashtra is 6 per 1000 population. The estimated prevalence of lower respiratory infection is 5 per 1000 population, and the estimated prevalence of low back pain is 7 per 1000 population, according to the Global Burden of Disease study.^[^ ^34^ ^]^ To calculate the sample size, with 95% confidence level and 95% precision and taking the mean of three diseases’ namely COPD, lower respiratory infection and low back pains prevalence, a sample size of 426 households was calculated. To cover for the loss because of non‐response it was decided to study 450 households.

Interview schedules with closed ended questions were used to collect the information. Primary data was collected on parameters like:

- Socioeconomic, demographic, and housing characteristics.
- Type and quantity of fuel used,
- The method of procurement and the type of stove used,
- The availability of a separate kitchen, its size and ventilation,
- Cooking practices and
- Time spent by the women while cooking (based on history and observation).

### 2.1 Data Analysis

Quantitative data: Binary logistic regression using IBM Statistical Package for the Social Sciences (SPSS) software package version 21 was used to analyze the data.

Logistic regression was used to predict the relationship between predictors (independent variables) and a predicted variable (the dependent variable). The variables for energy choices in binary logistic regression were used to study the effect of different factors on rural households’ energy choices. A logistic regression model was applied to determine the underlying socioeconomic factors like age, education, occupation, income, and fuel prices, which influence the adoption of clean fuels.

(1) $$\begin{array}{l} \ln\left(p\text{/}\left(1-p\right)\right)\;\;=\;\;a+BX\;\text{or}\;•p\text{/}\left(1-p\right)\;\;=\;\;ea+BX•p\text{/}\left(1-p\right)\;\;\\ \text{​}\text{​}\text{​}\;\;\;\;\;\;\;\;\;\;\;\;\;\;\;\;\;\;\;\;\;=\;\;ea\left(eB\right)X \end{array}$$

Where: “ln” is the natural logarithm,

logexp, where *e* = 2.71828

“*p*” is the probability that Y for cases equals 1,

*p* (*Y* = 1) “1 − *p*” is the probability that *Y* for cases equals 0,

1 − *p*(*Y* = 1) “*p*/(1 − *p*)” is the odds ln[*p*/1 − *p*] is the log odds, or “logit”

The following indicators, like age in years, type of house, type of kitchen, education of the respondent, the household's annual income, and spending on fuel, were included in the logistic regression model.

### 2.2 Dependent Variables

The logistic regression model has taken into account two different binary or dichotomous variables for two different energy sources. The first question asked to the respondents was whether they use the particular energy source or not, biomass versus non‐biomass users. Biomass includes firewood, cow dung, agricultural waste, and coal. Non‐biomass includes kerosene, LPG, and others, represented by “1” as a user and “0” otherwise. A further distinction was made by asking the type of fuel used for cooking and was further categorized into two different users as biomass and clean fuel. Biomass coded as “1” if the household is a frequent user and “0” for an occasional user. Similarly, the coding was done for the clean fuel user.

### 2.3 Independent Variables

Age: Categorized in four different groups, namely the lowest age group (<25 years), middle‐age group (25–34 years), upper‐middle age group (35–44 years), and the upper age group (>45 years).

Education: Education categorized into six different types, namely, illiterate, primary, middle, secondary, senior secondary, graduate, and higher.

Income: Income of rural households was classified into four different groups, viz. the lowest income group (<25 000), middle‐income group (25 001–35 000), upper‐middle‐income group (35 001–45 000) and the upper‐income group (>45 000)

Type of house: Respondents were inquired if they owned a house and further if it was puccae (concrete permanent structure), semi‐pucca (walls are concrete, and the roof is temporary) or a kutcha house (temporary structure).

Type of kitchen: The indoor cooking practice was dichotomized as in: a separate kitchen (cooking inside the house but in a different room used as a kitchen), no separate kitchen (cooking inside the house but in the absence of a separate kitchen), and outdoors.

Expenditure on fuel: Spending on any type of fuel, whether biomass or clean, was taken into account for analysis. Specifically, in the case of firewood, if it was collected or purchased was taken into account. When households collect firewood, they bear relatively high physical inconveniences without any costs for the firewood. Biomass was coded as “1” if the household bought firewood/clean fuel and “0” if they did not buy.

## 3 Results

### 3.1 Profile of the Area

The socioeconomic characteristics in all twenty villages were more or less similar with semi‐pucca houses. Education and healthcare facilities in the villages were satisfactory, with at least one primary school and one health center in each village. People in the block had an average standard of living, with most of them owning more than one modern household asset such as radio, electric fan, and television. Ownership of LPG stoves has also increased substantially since 2016.Women in the villages also shared the agriculture and animal husbandry workload.

Age: The respondent's age varied from 21 years to 55 years, with the mean age being 36 years.

Education: The majority of the respondents was literate, with 62.4% of the women having attended middle‐school.

Income: A quarter (27.1%) of households belonged to the lowest income category (**Table** **1**), 46.2% of the households were middle‐income households, and 26.7% were upper‐middle‐income households. The average annual per‐capita income was Rs 33 770/‐(≈450 USD).

**Table 1:** Socio‐economic and fuel characteristics of household

Variable		Number of of respondents (*n* = 450)	Percentage
Age of respondent	<25	71	15.8
	25–34	68	15.1
	35–44	112	24.9
	45 and above	199	44.2
Education level of respondent	Illiterate	39	8.7
	Primary	66	14.7
	Middle	281	62.4
	Secondary	16	3.6
	Senior secondary	40	8.9
	Graduate and Above	8	1.8
Occupation of respondent	Housewife	16	3.6
	Farmers/others	38	8.4
	Agricultural laborers	396	88.0
Income of the household	<25 000	122	27.1
	25 001–35 000	208	46.2
	35 001–45 000	120	26.7
	>45 000	450	100.0
Type of house	Pucca	98	21.8
	Semi‐pucca	189	42.0
	Kutcha	163	36.2
Separate kitchen in house	Yes	387	86.0
	No	63	14.0
Type of kitchen	Cooking inside living room	32	7.1
	Attached to main living area	356	79.1
	Semi‐open/Outside house	62	13.8
Type of fuel used^*)^	Biomass fuel	399	88.7
	Mixed fuel	229	50.9
	Other fuel	140	31.1
Most Frequently used fuel for cooking	LPG	236	52.4
	Wood	214	47.6
Reason for not using clean fuel	High cost of LPG refill	190	42.2
	Freely available biomass	193	42.9
	Distance traveled for the LPG refill	67	14.8

Note: *) Multiple answer.

### 3.2 Household Characteristics

The majority of the household surveyed was semi‐pucca (42%) and kutcha (36.2%) with tiles as a roof and generally poor to no ventilation. Walls of the houses were made of mud (49.3%) bricks (36.7%) with one (45.3%) or without windows (42.5%).

The average household size in the study villages was of six people. Most of the respondents were agricultural laborers. The majority of the households (86%) had a separate kitchen.

### 3.3 Fuel Characteristics

Physical access to LPG was 44.9% and half (50.9%) of the households used mixed fuels as the primary source (i.e., LPG and firewood, crop residue, dung cakes). Only 10% of the respondents were using LPG alone for their energy requirements. Out of the 202 households with an LPG connection, only 40 households (8.89%) obtained their LPG supplies under the PMUY scheme to provide clean fuel to the low‐income population. The most frequently used fuel for cooking was LPG (52.4%) combined with firewood (47.6%). The overall mean household consumption of firewood was 5 kg per day. Dung (21.6%) and kerosene (12.0%) were used less frequently. LPG was used for making tea and cooking vegetables, while chapattis were made on chulha. Wood was used by all the respondents for heating water during the winter season and making animal feed. Around 40.4% of the respondents gathered wood, whereas 27.3% bought it from the market. The majority of the respondents (88.0%) gathered fuel once or twice a week, with 67.6% respondents spending 2 to 3 h for gathering fuel. About 56.9% of women collected fuel, while men (40.4%) also contributed to the task.

### 3.4 Regression Analysis of Biomass Users versus Clean Fuel Users

Age: Logistic regression analysis of biomass users (firewood, agriculture residue, cow dung) indicated that in the older age group (35–45 years) respondents were twice likely to use biomass as compared to the younger age group (25–35 years). However, the youngest age group, below 25 years of age, used clean fuel, maybe they were aware of the adverse effects of smoke on health. Younger respondents were more likely to use LPG than the older age groups (**Table** **2**). However, there was a slight fluctuation in the oldest age group (of above 45 years). Similar results were also reported in a study conducted in Maharashtra's Buldhana district, where older women used traditional fuel more as a matter of habit than the younger women.^[^ ^35^ ^]^

**Table 2:** Logistic regression analysis of biomass/clean fuel users with socioeconomic characteristics

Variable	Biomass fuel				Clean fuel		
Age	Standard deviation	*p*‐Value	Odds ratio		Standard deviation	*p*‐Value	Odds ratio
<25	0.520	0.994	0.996		^a)^	0.162	
25–34	0.549	0.010	0.244^d)^		0.390	0.665	1.184
35–44	0.433	0.062	0.446		0.342	0.217	0.656
45>	^a)^				0.299	0.612	1.164
Education							
Illiterate	^a)^				^a)^	0.005	
Primary	6061.002	0.997	0.000		0.933	0.033	0.137
Middle	6061.002	0.997	0.000		0.891	0.213	0.329
Secondary	11 251.099	1.000	0.762		0.856	0.353	0.452
Senior Secondary	8427.682	1.000	1.469		1.128	0.018	0.070
Graduate and Above	15 285.552	1.000	2.678		0.914	0.648	0.659
Income							
<25 000	1.598	0.005	4.941^c)^		0.256	0.513	^a)^
25 001–35 000	0.897	0.028	2.451^d)^		0.297	0.555	1.163
35 001–45 000	^a)^					0.248	1.408
House Type							
Pucca	0.480	0.701	1.203		0.291	0.410	^a)^
Semi‐pucca	0.447	0.097	2.102		0.253	0.182	0.678
Kutcha	^a)^					0.461	0.830
Kitchen type							
No separate kitchen	0.767	0.043	0.212^d)^		0.491	0.574	1.318
Separate kitchen	0.484	0.338	1.589		0.311	0.956	0.983
Semi‐open/Open cooking		^a)^				^a)^	^a)^
No spending	0.481	0.000	7.570^b)^		0.306	0.006	
On LPG	0.447	0.285	1.613		0.247	0.009	2.237^c)^
All other ^a)^						0.527	0.855

^a)^ category; Robust standard errors in parenthesis

^b)^ Significant at 1%

^c)^ Significant at 5%

^d)^ Significant at 10%.

Education: The level of education was another significant factor associated with biomass fuel use, but in the study area, educated respondents were also using biomass fuel (Table 2).This indicated that the availability of free biomass influenced the users despite being educated.^[^ ^36^ ^]^ Respondents who were graduates and higher were more likely to use LPG (OR 0.65) than respondents with senior secondary level education (OR 0.07).

Income: There was a significant (*p* < 0.01) positive association between total annual household income and energy choice. The estimated coefficient, which represents the log of the odds ratio (OR), suggested that the odds of using firewood (collected or bought) were highest in the lower‐income group compared to other income groups. The logistic regression analysis revealed that those who have an annual income in the lowest income range (less than Rs 25 000), had more than four times higher probability of using biomass fuel (OR = 4.94) as compared to the higher income group (above Rs. 35 000). Those who had an annual income of Rs. 25 000 to 35 000 had more than two times higher probability of using biomass fuel (OR = 2.45) than the higher income group (above Rs 35 000). This was observed to be statistically significant, as well. The logistic regression analysis for clean fuel revealed that those who have an annual income of more than Rs 35 000 were having a higher probability of using LPG as fuel (OR = 1.40) as compared to the lower‐income group (Rs 25 000–35 000) (OR = 1.16). The income of the household had a significant effect on fuel choice. **Table** **3** highlights that lower‐income households are using lower quality fuels for energy needs. It shifts to a higher quality of fuels as the household income increases. It is gradually ascending in the “energy ladder,” and families in the middle‐income group are using all available energy options, indicating the stacking of fuel. In the study area, households often employed “multiple models” of stove and energy use, and the fuel change was observed to be partial.^[^ ^37^ ^]^ The conversion to clean fuels in the area remained slow. The likelihood of respondents not spending on the purchase of fuel was seven times higher than that of using biomass with an odds ratio of 7.570*** than respondents spending money on an LPG cylinder at the odds ratio of 1.61. The high cost of LPG limits its use thereby highlighting that economics determine the household fuel choice. These findings are consistent with the findings of other researchers.^[^ ^38^, ^39^, ^40^ ^]^

**Table 3:** Comparison of the mean values of energy adoption determinants

Variable	Description		Biomass user			Biomass non‐user			*p*‐Value
			No.	Percentage		No.	Percentage		
Type of house	Pucca		85	21.3%		13	25.5%		0.70847
	Semi‐pucca		170	42.6%		19	37.3%		
	Kutcha		144	36.1%		19	37.3%		
Age	<25		63	15.8%		8	15.7%		0.32
	25–34		59	14.8%		9	17.6%		
	35–44		95	23.8%		17	33.3%		
	>45		182	45.6%		17	33.3%		
Income	<25 000		117	29.3%		5	9.8%		0.00
	25 001–35 000		187	46.9%		21	41.2%		
	35 001–45 000		95	23.8%		25	49.0%		
	>45 000					0	0		
Spending on Fuel	No spending		184	46.1%		9	17.6%		0.000
	Cylinder		69	17.3%		15	29.4%		
	All others		146	36.6%		27	52.9%		

Separate kitchen: Households having separate kitchen were more likely to have clean fuel than other kitchen types. The odds of cooking within a living room were higher in households for biomass, with any level of formal education and older age groups (Table 3). In contrast, cooking in a separate kitchen was less likely to be found in a household that belonged to a lower income group, located in rural areas.

Expenditure on clean fuel: Spending on fuel that is clean fuel was twice the cost of biomass fuel with odds ratio of OR = 2.23 and OR = 0.85. Biomass fuel, being free of cost, was a significant factor of OR = 7.570 for the villagers who prefer not spending on clean fuel.

## 4 Discussion

This study assessed the behavior change in socioeconomic determinants of fuel choice and its markers, especially fuel types, cooking practices, socio‐demographic characteristics like education and income in rural areas of Nagpur district. Low household incomes, lack of a separate kitchen, preference for biomass due to free availability were observed as the cause for not using clean fuel. Around a fourth (27.1%) of rural households was agriculture laborers and belonged to the lowest income category. They used to cook in the living room area with no ventilation. Formal education was, surprisingly, a less significant factor. The results indicated that complete switching to clean fuel with an increase in income as described in the energy ladder had not taken place entirely in the study area because behavior change in the respondents has not taken place (despite ascending the income ladder in the first and second quartile of the income group). Clean fuel was made available before programs like PMUY but it did not scale‐up as the number of connections given were limited.^[^ ^41^ ^]^ Since Nagpur has 14 blocks, these numbers are less compared to other parts of India. The findings also indicated widespread fuel stacking, as shown by the works of other researchers.^[^ ^42^, ^43^ ^]^ As a result, the full benefits of clean fuels were not achieved. Conversion to cleaner fuels has remained slow due to cost being the limiting factor.

For the below the poverty line (BPL) population, spending Rs 700 per month (for clean fuel) is a considerable amount. Also, they do not get their subsidy until the cost of connection has been recovered. Many villagers found LPG too costly as 43.9% of the population uses biomass as it is freely available, and in any case, they have to burn it. The result corresponds to similar findings from the PMUY study done by another researcher^[^ ^44^ ^]^ and a clean cooking alliance study undertaken in Karnataka.^[^ ^45^ ^]^ The price of a non‐subsidized LPG cylinder was Rs 554 in Nagpur at the time of launch of PMUY in May 2016, but it rose to Rs 781.5 by the year 2018. Similarly, a subsidized cylinder's price rose from Rs 419.15 to Rs 499.48 in the same period. This also affected the affordability factor. Another reason for using firewood could be slow firewood burning. It is considered the best mode of cooking for some traditional recipes like biryani and non‐vegetarian curries, where the ingredients mix well and taste better. Slow cooking helps women who work in the field to mix all the ingredients and keep them to simmer. By the time they return from the field, the food is cooked. The smoke from chullah gives a smoky flavor to food and the firewood and cow dung being cheap and readily available.

The Petroleum Planning and Analysis Cell commissioned a study which has identified that the distance to the LPG distributor and long waiting time for a refill contributes as barrier to the adoption of LPG.^[^ ^44^ ^]^ The distributor's vehicle visits the area weekly. If the cylinder is somehow not refilled on that particular day, then the user may have to wait for the next eight days or get it refilled from block headquarters situated in Kondhali. In some villages, the connections are from other block headquarter (Katol), which does not have any distributors in the villages studied. As a result, the villagers have to get it refilled from block headquarters, which is cumbersome. Thus, the low accessibility of LPG also hampers its usage.

## 5 Conclusion

A complete transformation to cleaner cooking fuels is progressing at a slow pace in the district studied especially in rural areas. The present study highlights that though LPG connections have increased, the biomass is a vital fuel used in most households. The penetration of clean fuel was low with an increase in household income resulting in a shift of biomass from primary to secondary fuel. Free availability of biomass coupled with low income hindered the process of complete replacement to clean fuel. The authors hope that this study will help policymakers review, revise, and formulate a newer strategy to increase the program's reach and make it more extensive.

## Conflict of Interest

The authors declare no conflict of interest.
